# Circulating subpopulations of non-cytotoxic ILCs in diffuse large B-cell lymphoma

**DOI:** 10.1007/s00277-024-05831-8

**Published:** 2024-06-11

**Authors:** Agata Chwieduk, A. Smagur, M. Głowala-Kosińska, P. Borzdziłowska, W. Fidyk, I. Mitrus, M. Wilkiewicz, A. Hadryś, A. J. Cortez, S. Giebel

**Affiliations:** 1 Department of Bone Marrow Transplantation and Oncohematology, Maria Sklodowska-Curie National Research Institute of Oncology, Gliwice Branch, ul. Wybrzeze Armii Krajowej 15, Gliwice, 44-101 Poland; 2 Department of Biostatistics and Bioinformatics, Maria Sklodowska-Curie National Research Institute of Oncology, Gliwice Branch, Gliwice, Poland

**Keywords:** Innate lymphoid cells (ILCs), Cancer immunology, Diffuse large B-cell lymphoma

## Abstract

Non-cytotoxic innate lymphoid cells (ILCs) have been added to the list of immune cells that may contribute to the tumor microenvironment. Elevated levels of total ILCs and their subgroups have been reported in peripheral blood and tissue samples from patients with solid tumors, but their frequency in non-Hodgkin lymphomas, particularly diffuse large B-cell lymphoma (DLBCL), has not been clearly established. This study examined frequency and subset distribution in newly diagnosed DLBCL patients (nodal and extra-nodal) and compared it with blood specimens from healthy donors. The percentage of total ILCs (Lin − CD127+) was assessed by flow cytometry, as well as the four ILC subsets, defined as ILC1 (Lin − CD127 + cKit − CRTH2−), ILC2 (Lin − CD127 + cKit+/- CRTH2+), ILCp NCR- (Lin − CD127 + cKit + CRTH2- NKp46-) and NCR + ILC3 (Lin − CD127 + cKit + NKp46+). In the studied group of patients (*n* = 54), significantly lower levels of circulating total ILCs, ILC1, and ILCp NCR- were observed compared to the control group (*n* = 43). Similarly, there was a statistically significant decrease in the median frequency of NKp46 + ILC3 cells in lymphoma patients. Analysis of the ILC2 subpopulation showed no significant differences. The correlation of the distribution of individual subpopulations of ILCs with the stage and location of the tumor was also demonstrated. Our results suggest that circulating ILCs are activated and differentiated and/or differentially recruited to the lymph nodes or tumor microenvironment where they may be involved in antitumor defense. However, our observations require confirmation in functional studies.

## Introduction

The understanding of cancer development and tumor microenvironment was broadened nearly a decade ago by the recognition of the important role of innate lymphoid cells (ILCs). While the presence of some immune cells such as cytotoxic T cells is important for limiting tumor growth, the appearance of others, including tumor-associated macrophages, correlates with invasiveness, metastasis, and poor prognosis. The tumor microenvironment is often infiltrated by a wide variety of immune cells, including innate immune mediators such as NK cells, dendritic cells, and macrophages. Moreover, among the cells mediating adaptive immunity, the tumor microenvironment is infiltrated by helper CD4 + and cytotoxic CD8 + T and B lymphocytes. Adaptive immunity has been shown to play a critical role in the elimination of tumor cells, but we still know very little about the role of innate lymphocytes in this process. Cancer cells are a potential target for lymphocyte attack, resulting in the control of tumor progression [20]. The immune system response includes anticancer effects, but dysregulated inflammatory responses have also been linked to carcinogenesis and often precede tumor development [39]. In this way, the immune system not only protects the host from tumor development but may also promote the progression of pre-malignant cells to malignant cells. In addition to the classic cytotoxic natural killer (NK) cells and lymphoid tissue inducing (LTi) cells, the recently described non-cytotoxic ILC helper populations also belong to the innate lymphoid cells (ILCs) family [16]. ILCs, functionally resembling T cells in cytotoxicity and cytokine production, have emerged as important new effector cells of the immune system, also playing a key role in tumor immune surveillance, immune responses, and tissue homeostasis [26] [8]. Based on cytokine production characteristics, transcription factor expression profiles, and functional similarity with helper (Th1, Th2, and Th17) and cytotoxic T cell subsets, adult ILCs can be broadly classified into four lineages: conventional natural killer (cNK) and non-cytotoxic group 1, 2 and 3 ILCs [36]. Both ILC and innate cell-like lymphocytes (ILTC) are numerous tissue-resident lymphocytes that play a key role in many types of cancer. They respond to changes in the environment around them and act on a combination of innate and adaptive immunity, which makes them key coordinators of the ultimate anti-cancer immune response. These cells combine innate and adaptive immunity by initiating, enhancing, inhibiting, or suppressing the immune response in TME. Recent studies show that the ILC and ILTC subsets also share similar recruitment pathways for innate immune cells, such as macrophages or DCs, which are critical for the induction of strong adaptive antitumor immunity [25]. Group 1 innate lymphoid cells have a wide range of functions, including immunity to viruses and cancer cells, macrophage activation, cytotoxicity, and chronic inflammation. ILC1 express T-bet in response to interleukin-12 (IL-12), and secrete Th1 effector cytokines such as interferon gamma (IFN-γ) and tumor necrosis factor alpha (TNF-α) [10] [4]. Human ILC2 are defined by various cell surface markers and the expression of receptors for the cytokines IL-33 and IL-25. Their development and function depend on the GATA-binding protein 3 (GATA3) and the orphan receptor associated with the retinoic acid receptor-𝛼 (ROR𝛼) [1]. ILC2 abundantly secrete the type 2 cytokines IL-5, IL-4, and IL-13, and are strongly implicated with a microenvironment promoting tumor growth and blocking antitumor immunity [23]. ILC3 may promote tumor formation and progression by secreting IL-17, IL-22 and possibly by suppressing T-cell responses. ILC3 (Lin-CD127 + CD117+) comprises of two subsets of cells defined by their cell surface expression of natural cytotoxicity receptors (NCR). In humans, the NCR family consists of three molecules: NKp44 (NCR2, CD336), NKp46 (NCR1, CD335) and NKp30, the only NCR conserved in all mammals [32]. NKp46 and NKp44 are activating receptors that play a major role in tumor cell recognition and killing by NK cells [35] [21]. Analysis of their functional properties showed that NCR + ILC3 can promote the recruitment of other leukocytes to the tumor site by secreting chemotactic factors and activating tumor-associated angiogenesis [35]. An extensive analysis of ILC from human peripheral blood and tissue showed recently that ILCs with CD117 + phenotype, previously proposed to represent human ILC3, are enriched in multi-potent and uni-potent ILC precursors (ILCp) in the peripheral blood that can give rise to all know ILC subsets [18].

The role of ILCs in non-Hodgkin’s lymphomas (NHLs) has not been clarified yet. Hence, we studied the frequency and functionality of ILCs in DLBCL patients. Diffuse large B-cell lymphoma (DLBCL), the most common hematological type of malignancy among NHLs is heterogeneous in terms of biology, clinical presentation, and response to treatment [30]. To the best of our knowledge, this study performed the first analysis of ILC incidence and subset distribution of previously untreated DLBCL patients.

## Materials and methods

### Patients and healthy controls recruitment for the study

In the period of 24 months, 54 adult patients verified for DLBCL diagnosis in the Department of Bone Marrow Transplantation and Oncohematology (Maria Skłodowska-Curie National Research Institute of Oncology, Gliwice Branch), and 43 healthy donors were recruited. Of all of the enrolled patients, 30 had nodal lymphoma and 24 patients had extranodal DLBCL in the stomach, intestine, skin, etc. (Table 1). None of the patients had other coexisting tumors or pathologies affecting the immune system. The number of participants as well as all research procedures on material collection was approved by the Local Bioethics Committee. The criteria for exclusion of the participant from the study were age below 18, ECOG performance status > 2, bleeding diathesis with reduced plasma hemostasis parameters or PLT count (< 100G/l), pregnancy and other clinical symptoms and situations in which recruitment to the study could delay treatment.

**Table 1:** Clinical characteristics of DLBCL patients

*N*	54
Age (years)	59.9 (21–91)
Gender	
Male	23 (43%)
Female	31 (57%)
Clinical stage	
I - II	21 (39%)
III - IV	33 (61%)
International prognostic index (IPI)	
Low (0–1)	22 (41%)
Low-intermediate (2)	12 (22%)
High-intermediate (3)	12 (22%)
High (4–5)	8 (15%)
Localisation	
Nodal	30 (56%)
Extranodal	24 (44%)
DLBCL subtype	
None or single-hit	29 (54%)
Double-hit	9 (17%)
Triple-hit	13 (24%)
Unclassified	3 (6%)

### Immunophenotyping of innate lymphoid cells by flow cytometry

Freshly drawn blood, collected with the anticoagulant K_2_EDTA (BD-Plymouth, UK), was used for the cytometric detection of lymphoid cells in the circulation of patients and healthy donors. Whole blood staining with appropriate antibodies was performed within 3 h of material collection. The details of antibody staining are provided below as follows.

**Fig. 1:**
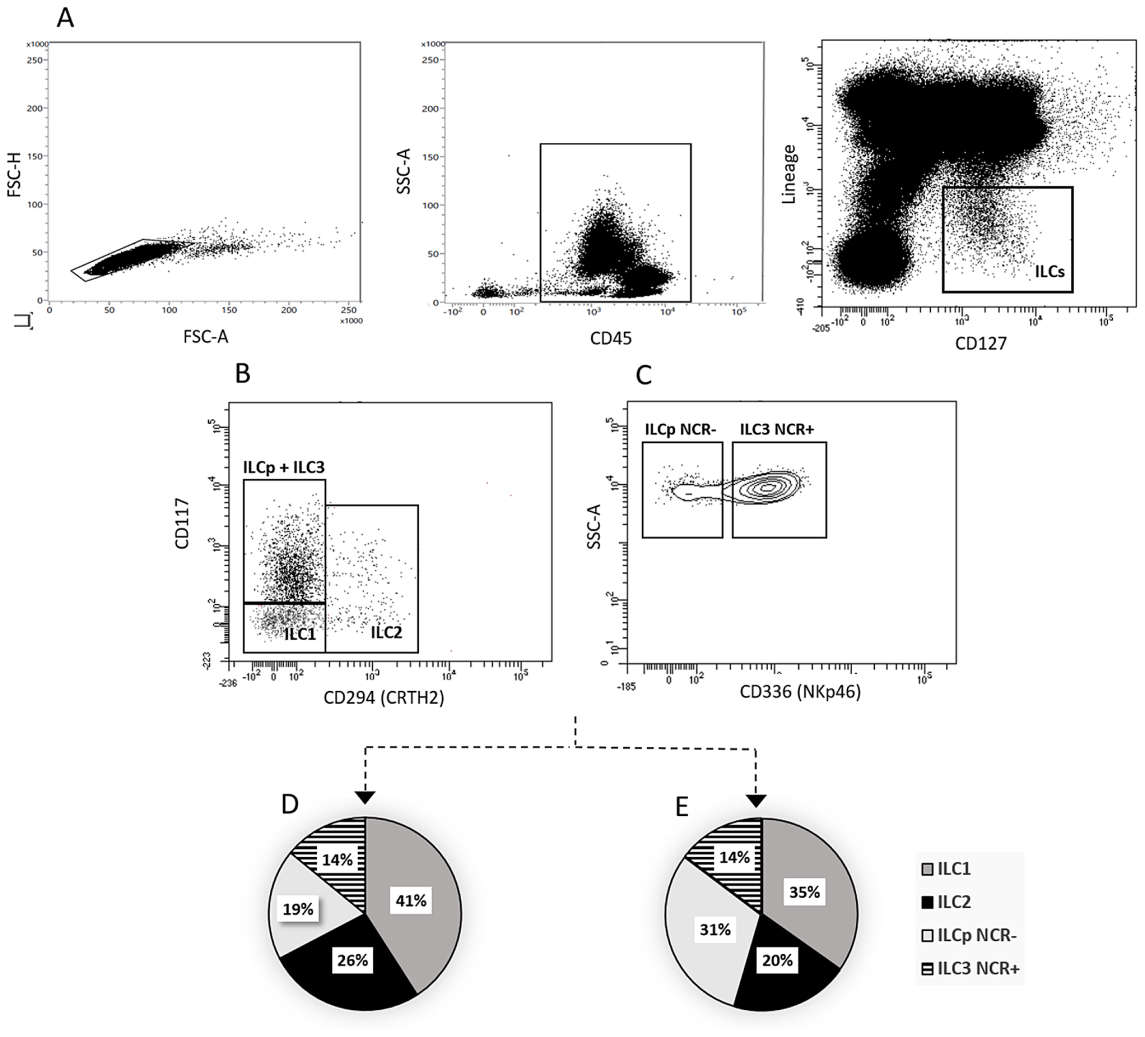
Innate lymphoid cells frequency and subtypes in peripheral blood of DLBCL patients at diagnosis and healthy donors. Firstly leukocytes and singlets were gated, then total circulating ILCs were identified as Lineage [anti-CD3 FITC mAb anti-CD4 FITC mAb, anti-CD8 FITC mAb anti-CD19 FITC mAb (Beckman Coulter), anti-CD34 FITC mAb, anti-CD14 FITC mAb, anti-CD15 FITC mAb (Becton Dickinson, San Jose, CA) and anti-CD20 FITC mAb, anti-CD123 FITC mAb, anti-CD TCRγδ FITC mAb, anti-CD16 FITC mAb]. For the identification of individual ILCs subpolulations were used: anti-CD127 PECy7 mAb, anti-CD117 BV421 mAb, anti-CD294 (CRTH2) APC-H7 mAb and anti-CD336 PerCP mAb (eBioscience, San Diego, CA). Representative example of the gating strategy to determine the absolute number (**A**) of total circulating ILCs and subtypes (**B**), by multiparameter flow cytometry. Expression of the natural cytotoxicity receptor (NCR) in the ILC3 subpopulation (**C**). Average percentages of total ILCs and their subpopulations in DLBCL (**D**), and healthy donors (**E**)

For ILC analysis, peripheral blood mononuclear cells (PBMC) were separated from whole blood samples by density gradient centrifugation using Ficoll Paque Plus (GE Healthcare, Uppsala, Sweden). Cells were then washed and resuspended in phosphate-buffered saline (PBS). To study cell division by flow cytometry 2 × 10^6^ PBMCs were labeled with the following mouse anti-human monoclonal antibodies. ILC were defined as CD45 + cells, which were gated on forward scatter height (FSC-H) vs. FSC area (FSC-A) to exclude doublets. Lineage cocktail (Lin) for the depletion of T cells, B cells, NK cells, monocytes, granulocytes, basophils, DC, and hematopoietic progenitor cells included: [anti-CD3 FITC mAb, anti-CD4 FITC mAb, anti-CD8 FITC mAb, anti-CD19 FITC mAb (Beckman Coulter, Brea, CA), anti-CD34 FITC mAb, anti-CD14 FITC mAb, anti-CD15 FITC mAb (Becton Dickinson, San Jose, CA) and anti-CD20 FITC mAb, anti-CD123 FITC mAb, anti-CD TCRγδ FITC mAb, anti-CD16 FITC mAb (BioLegend, San Diego, CA). For the identification of individual ILC subpopulations following antibodies were used: anti-CD127 PECy7 mAb, anti-CD117 BV421 mAb, anti-CD294 (CRTH2) APC-H7 mAb, and anti-CD336 PerCP mAb (eBioscience, San Diego, CA). At least 10^6^ MNCs were analyzed on an eight-color FACSCanto II flow cytometer (BD Biosciences, San Jose, CA). Data was analyzed using FacsDiva 9 software (BD Biosciences, San Jose, CA). Innate lymphoid cells defined as Lin-CD127 + were calculated as the absolute number (x10^6^/L) in the peripheral blood and percentage among leukocytes (WBC). ILCs subpopulations (ILC1, ILC2, ILCp NCR-, ILC3 NCR+) were calculated as both leukocyte percentage and total blood ILC (ILCtot). The gating strategy is shown in Fig. 1. Cell populations were identified based on isotype controls.

Based on the literature, we determined that a lineage mix useful for excluding rare populations from the ILC gate should contain at least antibodies for CD3, CD4, CD8, CD14, CD15, CD16, CD19, CD20, CD33, CD34, CD203c, and FcɛRI detection [33]. Regarding the phenotype of the three different human ILC subsets, namely ILC1, ILC2, and ILC3. ILC1 is known to have the immunophenotype CRTH2-cKit (CD117)-CD56-, ILC2 is CRTH2 + cKit(CD117)+/-CD56-, while ILC3 is CRTH2-cKit(CD117) + CD56+/− [29] [28]. However, the work of Nagasawa and Chen indicate that CD56 is expressed by some ILCp and ILC2 [6] [22] [7]. Also, the latest work of Trabanelli et al. indicates that the CD56 should not be used in the lineage cocktail being expressed on a subset of ILC3 [33]. The selection of the appropriate natural cytotoxicity receptor (NCR) used for ILC3 NCR + identification presented a challenge. In humans, the NCR family consists of three molecules: NKp30, NKp44, and NKp46. However, only NKp46 is present in both humans and mice and is expressed regardless of the activation status of the cells. We, therefore, decided to use NKp46 [11] [32].

### Statistical methods

Differences in peripheral blood concentrations of individual ILC populations between the DLBCL group and the control group and between subgroups of patients were analyzed using the U Mann-Whitney test. Correlations between the total ILC in peripheral blood, including numerous their and other quantitative variables were analyzed using the Spearman correlation test. The Fisher Z-Transformation was used to compare Spearman correlation coefficients between the two experimental groups for each analysis. Differences and correlations with *p* < 0.05 were considered statistically significant. Statistical analysis was performed using STATISTICA version 13.3 software (StatSoft Inc., Tulsa, OK, USA).

## Results

### Characteristics of the study population

We compared the frequency of total ILCs and ILC subsets distribution in PB samples from 43 healthy subjects and 54 newly diagnosed DLBCL patients [(median age 59.9, range 21–91; sex ratio (M: F) 23:31; primary DLBCL 54 of 54 (100%); cytogenetic risk group: low/intermediate 29 of 54 (54%), high (double- and triple-hit) 22 of 54 (41%)]. The patients were divided into groups that differed in the stage of the disease, localization of the lymphoma, and in terms of rearrangements in the *MYC, BCL2*, and *BCL6* genes (Table 1). The analysis showed a clear distinction between the percentage of complete ILCs and some subsets of ILCs in patients and healthy donors. The peripheral blood morphology of patients and healthy donors showed no differences among leukocytosis. But, the absolute number (x10^3^/ul) of normal lymphocytes was significantly reduced in the DLBCL group [1.68 × 10^6^/L (0.60–2.79)] vs. [2.32 × 10^6^/L (0.77–4.90)] (*p* < 0.001). However, there were no differences in the number of leukocytes or lymphocytes between the studied subgroups of patients.

### ILCs and their subpopulations in DLBCL patients and healthy controls

**Fig. 2:**
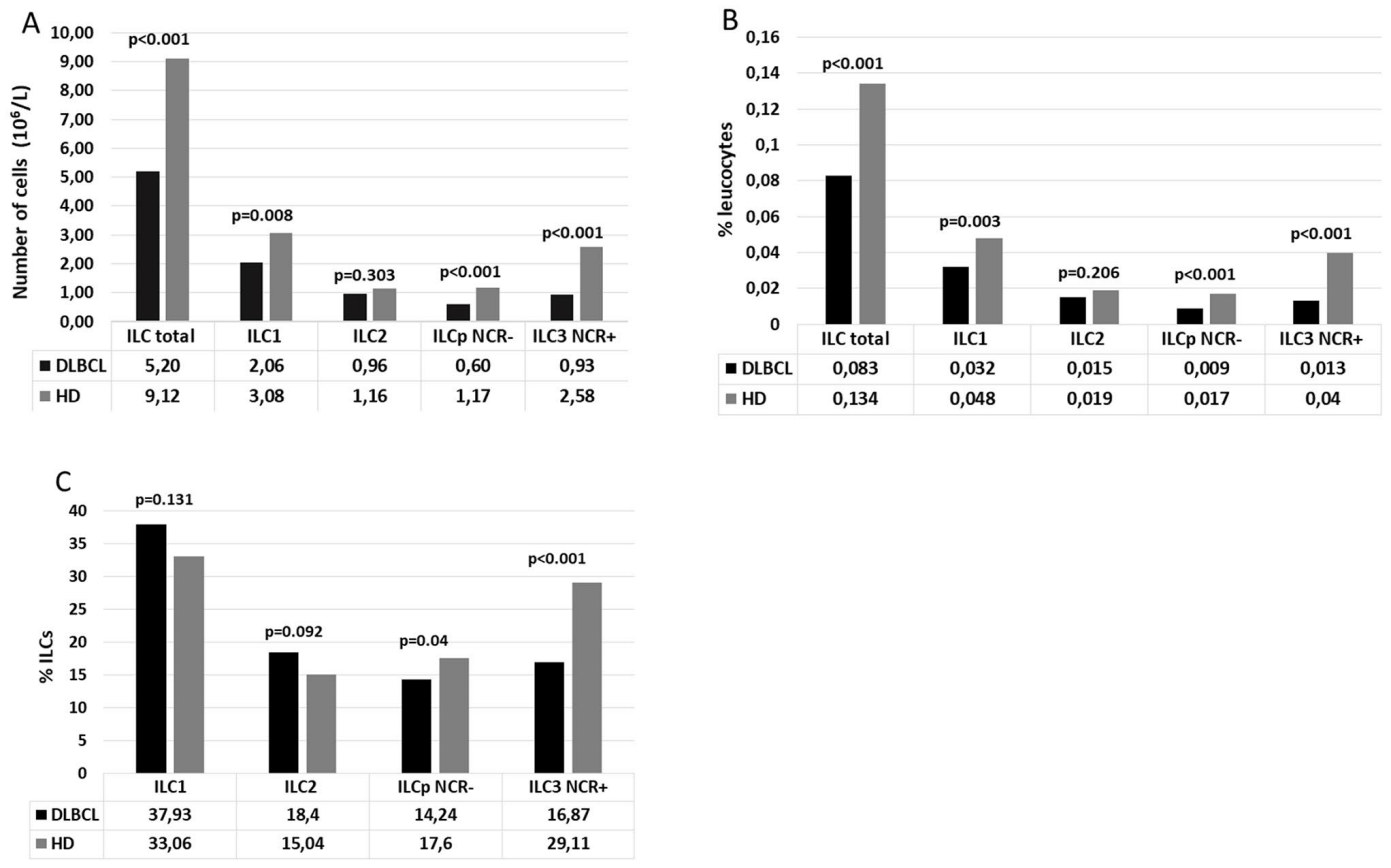
Total ILC frequencies and distribution of their subsets in PB samples from patients (DLBCL) (median, *n* = 54) vs. healthy donors (HD) (median, *n* = 47), absolute number (x10^6^/L) (**A**), percentages among leukocytes (**B**) and total ILCs (**C**). The normality of the data distribution was tested using the Shapiro-Wilk test. Statistical significance was tested using Wilcoxon (MannWhitney) non-parametric test if the data were not-normally distributed, otherwise unpaired t-test was used

The absolute number of Lin-CD127 + ILCs was found to be significantly lower (*p* < 0.001) in DLBCL patients [5.201 × 10^6^/L (1.391–30.356)] than in healthy donors [9.120 × 10^6^/L (1.492–27.006)]. Similarly, circulating ILC1 [2.057 × 10^6^/L (0.318–26.643)] vs. [3.075 × 10^6^/L (0.437–12.036)] (*p* = 0.003), ILCp NCR- [0.75 × 10^6^/L (0.065–2.777)] vs. [1.565 × 10^6^/L (0.105–8.431)] (*p* < 0.001) and ILC3 NCR + rates [0.929 × 106/L (0.03–6.689)] vs. [2.581 × 106/L (0.148–9.435)] (*p* < 0.001) were decreased in PB of DLBCL patients. Among all ILC subgroups tested, there were no statistical differences only for ILC2 as total cell count (*p* = 0.303) [0.964 × 10^6^/L (0.043–8.943)] vs. [1.156 × 10^6^/L (0.193–10.146)] and as a percentage of leukocytes [0.015% (0.0003–0.105) vs. 0.019% (0.006–0.151)] (*p* = 0.206). Similarly, the rate of type 2 ILC among total ILC did not differ [18.4% (2.9–88.1) vs. 15.0% (4.9–64.6)] (*p* = 0.092) compared to the control group. (Fig. 2). Also, the presence of the natural cytotoxicity receptor (NCR) in the ILC3 subpopulation was significantly higher in healthy donors, both as total cell count among leukocytes and relative to the total number of ILCs (*p* < 0.001 in three analyses) (Fig. 2).

**Fig. 3:**
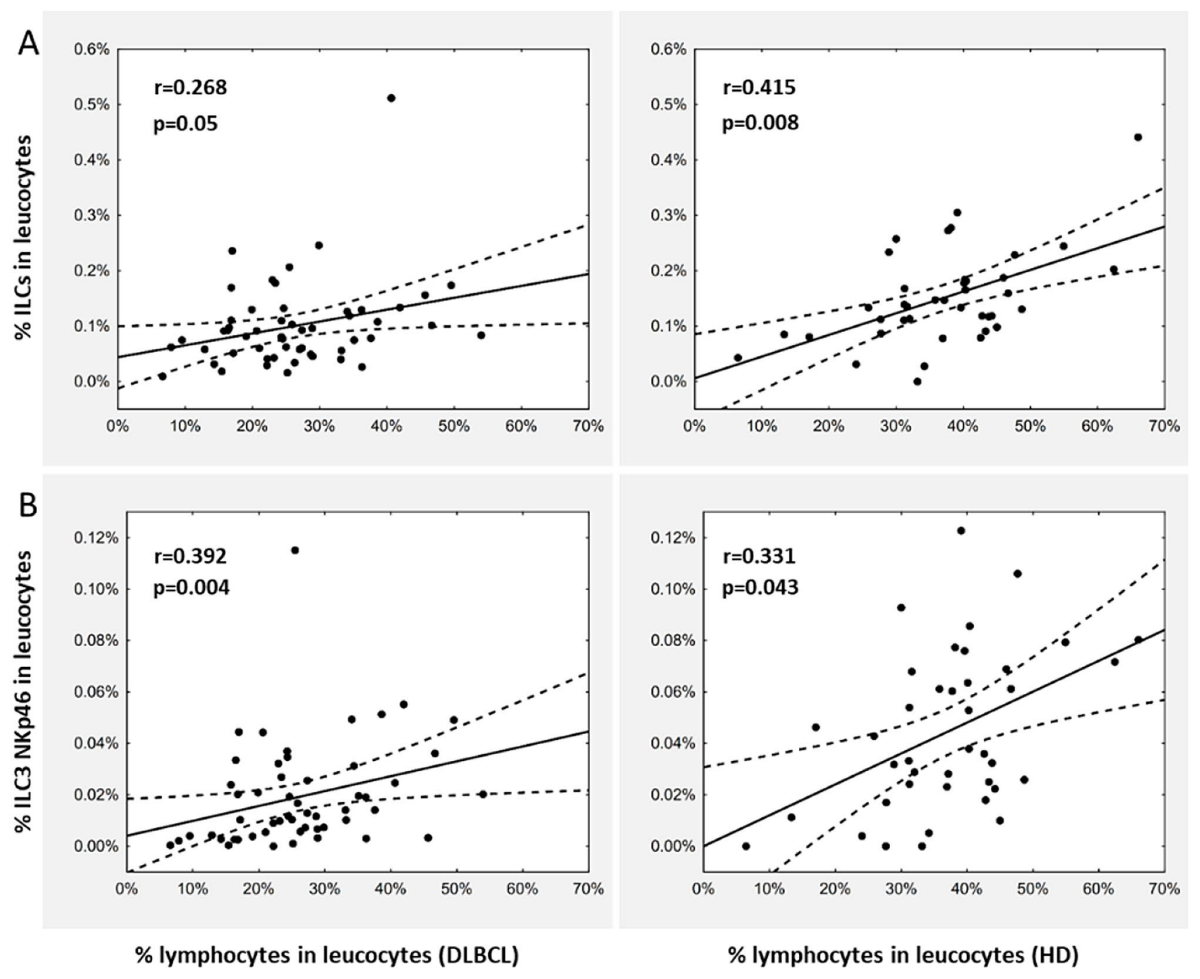
Correlation between innate lymphoid cells (ILCs) frequency and lymphocytes (**A**) and between ILC NKp46 and lymphocytes (**B**) in peripheral blood of DLBCL patients at diagnosis and healthy donors (HD). Statistical analyzes were performed using Spearman’s rho correlation coefficient analysis

Furthermore, analysis of Spearman’s rank coefficient of correlation between the percentage of total ILCs and the number of lymphocytes showed a low positive correlation in both DLBCL patients and healthy donors (Fig. 3A). A similar correlation was observed for the percentage of ILC3 NKp46 (Fig. 3B). However, Fisher Z-Transformation showed no statistically significant differences between correlations in diseased and healthy groups for both total ILC (*p* = 0.757) and ILC3 NKp46 (*p* = 0.453) (data not shown).

### ILCs and their subpopulations according to DLBCL subgroups

Among DLBCL patients ILCs and their subpopulations were analyzed according to the presence of extranodal lesions and clinical stage, taking into account the IPI (International Prognostic Index).

In the group of 54 patients with DLBCL, no significant relationship was observed between the absolute numbers circulating ILC Lin-CD127+, as well as the percentage of these cells among peripheral blood leukocytes and the presence or absence of extranodal locations. The distribution of the ILC subpopulation was also not associated with these clinical features. Only in relation to the total number of ILC, the percentage of ILC3 NCR + showed statistically significant differences (Table 2).

**Table 2:** Frequencies of total ILCs and their subpopulations in blood samples from patients with DLBCL patients of different clinical stages (I/II vs. III/IV)

	Clinical stage I -II (*N* = 21)	Clinical stage III - IV (*N* = 32)	*p*
ILCs total (% leucocytes)	0.096 (0.031–0.512)	0.0622 (0.009–0.024)	**= 0.028**
ILC1 (Lin- CD127 + CD117- CD294-) (% leucocytes)	0.038 (0.014–0.449)	0.025 (0.003–0.099)	**= 0.013**
ILC2 (Lin- CD127 + CD117 + CD294+) (% leucocytes)	0.013 (0.005–0.105)	0.018 (0.0003–0.091)	= 0.971
ILCp NCR- (Lin- CD127 + CD117 + CD294- NKp46-) (% leucocytes)	0.014 (0.002–0.045)	0.010 (0.001–0.035)	= 0.08
NCR + ILC3 (Lin- CD127 + CD117 + CD294-) (% leucocytes)	0.01 (0.0003–0.049)	0.017 (0.002–0.115)	= 0.059
ILC1 (Lin- CD127 + CD117- CD294-) (% ILCs)	37.76 (16.01–88.42)	38.31 (12.03–81.11)	= 0.445
ILC2 (Lin- CD127 + CD117 + CD294+) (% ILCs)	15.74 (5.38–61.89)	26.62 (2.93–88.12)	**= 0.049**
ILCp NCR- (Lin- CD127 + CD117 + CD294- NKp46-) (% ILCs)	12.44 (1.16–32.94)	14.01 (3.10–55.31)	= 0.525
NCR + ILC3 (Lin- CD127 + CD117 + CD294- NKp46-) (% ILCs)	20.15 (1.50–55.70)	16.45 (1.88–47.49)	= 0.398
ILCs total (x10^6^/l)	6.135 (3.171–30.357)	4.586 (1.391–15.552)	**= 0.048**
ILC1 (Lin- CD127 + CD117- CD294-) (x10^6^/l)	2.177 (0.859–26.644)	1.749 (0.318–6.492)	**= 0.046**
ILC2 (Lin- CD127 + CD117 + CD294+) (x10^6^/l)	0.927 (0.282–8.944)	1.090 (0.043–6.961)	= 0.682
ILCp NCR- (Lin- CD127 + CD117 + CD294- NKp46-) (x10^6^/l)	0.812 (0.185–2.637)	0.709 (0.065–2.777)	= 0.615
NCR + ILC3 (Lin- CD127 + CD117 + CD294-) (x10^6^/l)	1.067 (0.162–6.689)	0.715 (0.031–4.553)	= 0.124

The analysis revealed quantitative differences in the ILCtot and type 1 ILC as an absolute number in PB and percentage among leukocytes (Table 3). Additionally, we noticed a statistically significant increase in the percentage of ILC2 in relation to the total number of ILCs (*p* = 0.049) between patients in various clinical stages (I/II vs. III/IV) (*n* = 54) (Table 3). However, Spearman’s rank correlation coefficient analysis for ILC2% showed no statistically significant correlation with International Prognostic Index risk groups [rho = 0.174, (*p* = 0.208)].

**Table 3:** Frequencies of total ILCs and its subpopulations in blood samples from patients with nodal DLBCL vs. extranodal DLBCL

	Nodal (*N* = 30)	Extranodal (*N* = 24)	*p*
ILCs total (% leucocytes)	0.088 (0.010–0.512)	0.079 (0.027–0.236)	= 0.923
ILC1 (Lin- CD127 + CD117- CD294-) (% leucocytes)	0.032 (0.003–0.449)	0.027 (0.010–0.138)	= 0.734
ILC2 (Lin- CD127 + CD117 + CD294+) (% leucocytes)	0.018 (0.0003–0.105)	0.012 (0.003–0.091)	= 0.161
ILCp NCR- (Lin- CD127 + CD117 + CD294- NKp46-) (% leucocytes)	0.011 (0.002–0.044)	0.013 (0.001–0.045)	= 0.939
NCR + ILC3 (Lin- CD127 + CD117 + CD294- NKp46+) (% leucocytes)	0.010 (0.0003–0.049)	0.017 (0.002–0.115)	= 0.059
ILC1 (Lin- CD127 + CD117- CD294-) (% ILCs)	38.03 (12.03–87.77)	36.60 (16.01–88.42)	= 0.536
ILC2 (Lin- CD127 + CD117 + CD294+) (% ILCs)	27.99 (2.93–88.12)	17.27 (5.38–50.57)	= 0.146
ILCp NCR- (Lin- CD127 + CD117 + CD294- NKp46-) (% ILCs)	12.97 (1.16–55.31)	13.90 (3.10–32.94)	= 0.263
NCR + ILC3 (Lin- CD127 + CD117 + CD294- NKp46+) (% ILCs)	9.11 (1.50–48.32)	22.18 (2.12–55.70)	= **0.005**
ILCs total (x10^6^/l)	5.525 (1.406–30.357)	4.881 (1.391–15.552)	= 0.503
ILC1 (Lin- CD127 + CD117- CD294-) (x10^6^/l)	2.207 (0.318–26.644)	1.766 (0.653–8.567)	= 0.264
ILC2 (Lin- CD127 + CD117 + CD294+) (x10^6^/l)	1.449 (0.043–8.944)	0.767 (0.183–6.961)	= 0.050
ILCp NCR- (Lin- CD127 + CD117 + CD294- NKp46-) (x10^6^/l)	0.766 (0.110–2.776)	0.730 (0.065–2.638)	= 0.327
NCR + ILC3 (Lin- CD127 + CD117 + CD294- NKp46+) (x10^6^/l)	0.809 (0.031–3.328)	0.989 (0.155–6.689)	= 0.155

Both proportions of Lin-CD127 + cells showed no difference in the groups of patients divided by DLBCL subtype (double-hit vs. triple-hit) (data not shown). The only association between the percentage of cells tested and the subtype (double hit vs. triple hit) of lymphoma identified in patients was observed for ILC3 NCR+ [0.044% (0.004–0.055) vs. 0.01% (0.000–0.031)] (*p* = 0.023) among leukocytes and in the percentage of ILC3 NCR+ [28.24% (4.7–47.5) vs. 14.6% (1.5–26.3)] (*p* = 0.016) among all ILC (data not shown).

## Discussion

The growing number of studies on ILCs allows us to gain a broader knowledge of how the responses of these cells are shaped by particular tissue types and specific disease signals. The need for better understanding of ILCs biological roles is reflected in scientific studies describing their contradictory roles in cancer, inflammation, and immunopathology. So far, the knowledge of the role of ILCs in cancer has been based mainly on studies in solid tumors. Our research will, at least to a small extent, fill the gap in understanding the role of ILC in NHL lymphomas. B-cell non-Hodgkin’s lymphomas are a heterogeneous group of lymphoproliferative diseases characterized by diverse histology, complex clinical picture, and variable prognosis. Within B-cell lymphomas, different subtypes arise from different stages of B-cell differentiation, and different mechanisms may be involved in its pathogenesis [13]. However, data on ILC incidence and subgroup distribution of previously untreated DLBCL patients are not available to date, and the analysis of innate lymphoid cells in hematologic malignancies is still in its infancy. Our results are partly consistent with two previous reports indicating rather reduced or unchanged levels in peripheral blood in patients with hematological malignancies.

As far as we know, only one paper deals with the distribution and phenotype of both NK and ILC in the PB of NHL patients. No differences between healthy donors and NHL patients, either in terms of ILC frequency or subset distribution, have been observed. However, total ILC in the PB of NHL patients showed a slight but significant decrease in PD-1 and CD62L expression and a concomitant increase in activation/functional markers such as CD69, granzyme B, and perforin, suggesting a potential involvement of ILC in a direct antitumor effect [24]. In vitro experiments have shown that neoplastic cells (NHL) affect the activation of ILC, the phenotype of which may vary depending on the NHL cell line used and the presence of other immune cells. These results suggest that the immunosuppressive microenvironment created by the tumor may negatively regulate ILC. In addition, it has been shown that the presence of Tregs can inhibit the cytotoxic functions of ILCs and potentially reduce their antitumor activity in NHL patients.

However, analyses carried out by de Weerdt et al. involving the frequency, distribution, and functionality of ILCs in the peripheral blood of lymphocytic leukemia (CLL) patients compared with healthy controls demonstrated different results. They showed both a significant increase in the number of ILCs and a positive correlation of the number of ILCs with the number of lymphocytes in the patient group. This suggests a link between the increase in ILC and disease progression [9] and may be due to the increased lymphocytosis found in CLL patients. Unlike CLL, DLBCL patients have significantly lower peripheral blood lymphocyte counts compared to healthy controls. Our studies also showed a positive correlation between ILC and lymphocyte percentages in both healthy and diseased groups. However, statistically lower lymphocyte levels and an associated reduction in ILC counts in DLBCL patients has been observed.

Group 2 of ILCs are mostly considered as a subset that promotes the development of tumors, since the type 2 cytokines that they produce are associated with tumor growth and blocking of the antitumor immune response in tumor microenvironments (TMEs) [19]. High expression of ILC2 has been detected in solid tumors such as breast, lung, prostate, and gastric cancer, as the IL-33 produced by them is the main activator of ILC2 and usually promotes tumor growth, angiogenesis, and metastasis [14] [38] [5]. Some reports indicate that a high level of ILC2 has a positive effect on the Tregs population, which impairs the antitumor T-cell response and is usually associated with poor prognosis and higher tumor staging [31]. In our study, we also found a positive correlation between the level of ILC2 and the stage of the lymphoma. The analysis of circulating ILCs showed a statistically significant increase in the percentage of group 2 ILCs in patients with a higher (III/IV) stage of the disease, which may suggest a similar effect on lymphoma TME immunity as in the cited studies. The results presented a lack of correlation between ILC2 and IPI which may result from unequal or too small representation of individual risk groups. Further studies, especially prospective randomized clinical trials are required to clearly resolve this issue.

Trabanelli et al. performed ILCs research on patients with acute myeloid leukemia (AML). Similarly to our results, they showed a very significant reduction in circulating ILC in patients at the onset of the disease compared to controls, both in percentage and in absolute numbers. However, the analyzed relative frequency of different ILC subsets showed a significant enrichment of ILC1 in AML patients but also a concomitant high reduction of ILC3 NCR + cells. No differences were detected in the levels of ILC2 and ILC3 NCR- cells. There was also no difference in either the frequency of total circulating ILCs or the distribution of ILC subsets across cytogenetic risk groups. Moreover, when comparing peripheral blood (PB) and bone marrow (BM), no differences were found in either the percentage of total ILCs or in the relative proportions of the different ILC subsets, suggesting a similar composition of ILCs in BM and PB in patients with AML at disease onset [32]. In addition, they showed that ILCs were partially restored in patients responding to therapy. While ILC1 was still elevated compared to healthy donors, the total number of ILC and ILC3 NCR + cells returned to normal levels. A group studying innate lymphoid cells in inflamed intestinal tissues of patients with Crohn’s disease showed that the differentiation of ILC3 to CD127(+) ILC1 is reversible in vitro and in vivo. CD127(+) ILC1 differentiated into ILC3 in the presence of interleukin-2 (IL-2), IL-23, and IL-1β depending on the transcription factor RORγt [3]. These observations suggest that environmental factors determine the composition, function, and phenotype of ILC1 and ILC3 in the intestine. It is possible that a similar process occurs with AML in successfully treated patients. The results of the current study suggest that adult ILC3s self-renew or are replenished from tissue-resident progenitors, particularly RORγt + progenitors, which can give rise to mature ILC3s in both mice and humans [12]. Studies performed on intestinal tissue material have shown that NKp46-CCR6-RORγt + ILC3 precursors give rise to NCR + ILC3 and, to a lesser extent, an LTi-like subpopulation of ILC3, in a process regulated by local tissue cytokines [15] [34]. The specific ILC3 subpopulations in adults vary depending on the location of the lymphoid tissue. Unlike ILC3 NCR-, which are largely restricted to lymphoid structures such as mesenteric lymph nodes and cryptopathies in the small intestine and colon, NCR + ILC3 are excluded from lymph nodes but can be found both in the lamina propria and in isolated follicles lymphatic cells (ILF) in the small intestine [27]. Local tissue signals likely enable the maintenance and survival of specific ILC3 subpopulations in specific tissue microenvironments [37]. The group of extranodal DLBCLs we studied has shown variability in terms of tumor locatization. Therefore, despite a significant increase in the percentage of ILC3 NCR + in ILC subpopulations in the extranodal group, the cause of this increase in only 54 patients cannot be clearly determined. Additionally, the increase in NKp46 + ILC3 was only observed as a percentage of total circulating ILCs, not as a total number or percentage among leukocytes, which may be due to the plasticity of ILCs.

Ongoing research on ILC plasticity promises to advance knowledge on the transformation of mature ILCs into other subtypes compared to the directed development of ILC precursors (ILCp) into specific ILC populations. Future studies should also outline the similarities in both NK and ILC dysfunction in various hematologic malignancies to identify similarities and determine whether these defects serve as major contributors to antitumor immunity evasion [17].

Oncology treatment entered the era of immunotherapy some time ago, therefore it seems necessary to understand the plasticity of ILCs and their impact on tumor immunology. The biggest problem discovered so far with NK cell-based immunotherapies is whether the NK cells transferred to cancer patients will develop into tumor-promoting ILC1-like cells. It is also possible that cancer immunotherapy could benefit from exploiting the plasticity of ILC [2].

Our findings establish baseline reference values for ILC frequency and function, as well as the distribution of ILC subtypes in DLBCL patients at the time of diagnosis. These values may provide a useful benchmark for assessing the effect of anti-cancer treatment on ILC number or function. However, the importance of ILC dysregulation and functional impairment in B-NHL requires further investigation.

## Data Availability

The data that support the findings of this study are not openly available due to reasons of sensitivity and are available from the corresponding author upon reasonable request. Data are located in controlled access data storage at Department of Bone Marrow Transplantation and Oncohematology, Maria Sklodowska-Curie National Research Institute of Oncology, Gliwice branch, Gliwice, Poland

## References

[1] Bal SM, Bernink JH, Nagasawa M, Groot J, Shikhagaie MM, Golebski K, van Drunen CM, Lutter R, Jonkers RE, Hombrink P, Bruchard M, Villaudy J, Munneke JM, Fokkens W, Erjefält JS, Spits H, Ros XR (2016) IL-1β, IL-4 and IL-12 control the fate of group 2 innate lymphoid cells in human airway inflammation in the lungs. Nat Immunol 17:636–645. 10.1038/ni.3444 27111145 10.1038/ni.3444

[2] Bald T, Wagner M, Gao Y, Koyasu S, Smyth MJ (2019) Hide and seek: plasticity of innate lymphoid cells in cancer. Semin Immunol 41:101273. 10.1016/j.smim.2019.04.001 30979591 10.1016/j.smim.2019.04.001

[3] Bernink JH, Krabbendam L, Germar K, de Jong E, Gronke K, Kofoed-Nielsen M, Munneke JM, Hazenberg MD, Villaudy J, Buskens CJ, Bemelman WA, Diefenbach A, Blom B, Spits H (2015) Interleukin-12 and – 23 control plasticity of CD127 + group 1 and Group 3 Innate Lymphoid Cells in the Intestinal Lamina Propria. Immunity 43:146–160. 10.1016/j.immuni.2015.06.019 26187413 10.1016/j.immuni.2015.06.019

[4] Bernink JH, Peters CP, Munneke M, te Velde AA, Meijer SL, Weijer K, Hreggvidsdottir HS, Heinsbroek SE, Legrand N, Buskens CJ, Bemelman WA, Mjösberg JM, Spits H (2013) Human type 1 innate lymphoid cells accumulate in inflamed mucosal tissues. Nat Immunol 14:221–229. 10.1038/ni.2534 23334791 10.1038/ni.2534

[5] Bie Q, Zhang P, Su Z, Zheng D, Ying X, Wu Y, Yang H, Chen D, Wang S, Xu H (2014) Polarization of ILC2s in Peripheral Blood might contribute to Immunosuppressive Microenvironment in patients with gastric Cancer. J Immunol Res 2014:1–10. 10.1155/2014/923135 10.1155/2014/923135 PMC3987940 24741632

[6] Calvi M, Di Vito C, Frigo A, Trabanelli S, Jandus C, Mavilio D (2022) Development of human ILCs and impact of unconventional cytotoxic subsets in the pathophysiology of inflammatory diseases and Cancer. Front Immunol 13:914266. 10.3389/fimmu.2022.914266 35720280 10.3389/fimmu.2022.914266 PMC9204637

[7] Chen L, Youssef Y, Robinson C, Ernst GF, Carson MY, Young KA, Scoville SD, Zhang X, Harris R, Sekhri P, Mansour AG, Chan WK, Nalin AP, Mao HC, Hughes T, Mace EM, Pan Y, Rustagi N, Chatterjee SS, Gunaratne PH, Behbehani GK, Mundy-Bosse BL, Caligiuri MA, Freud AG (2018) CD56 Expression Marks Human Group 2 Innate Lymphoid Cell Divergence from a Shared NK Cell and Group 3 Innate Lymphoid Cell Developmental Pathway. Immunity 49:464–476e4. 10.1016/j.immuni.2018.08.010 30193847 10.1016/j.immuni.2018.08.010 PMC6148384

[8] Dadi S, Chhangawala S, Whitlock BM, Franklin RA, Luo CT, Oh SA, Toure A, Pritykin Y, Huse M, Leslie CS, Li MO (2016) Cancer Immunosurveillance by tissue-resident innate lymphoid cells and innate-like T cells. Cell 164:365–377. 10.1016/j.cell.2016.01.002 26806130 10.1016/j.cell.2016.01.002 PMC4733424

[9] de Weerdt I, van Hoeven V, Munneke JM, Endstra S, Hofland T, Hazenberg MD, Kater AP (2016) Innate lymphoid cells are expanded and functionally altered in chronic lymphocytic leukemia. Haematologica 101:e461–e464. 10.3324/haematol.2016.144725 27662009 10.3324/haematol.2016.144725 PMC5394863

[10] Fuchs A, Vermi W, Lee JS, Lonardi S, Gilfillan S, Newberry RD, Cella M, Colonna M (2013) Intraepithelial type 1 innate lymphoid cells are a unique subset of IL-12- and IL-15-Responsive IFN-γ-Producing cells. Immunity 38:769–781. 10.1016/j.immuni.2013.02.010 23453631 10.1016/j.immuni.2013.02.010 PMC3634355

[11] Galon J, Mlecnik B, Bindea G, Angell HK, Berger A, Lagorce C, Lugli A, Zlobec I, Hartmann A, Bifulco C, Nagtegaal ID, Palmqvist R, Masucci GV, Botti G, Tatangelo F, Delrio P, Maio M, Laghi L, Grizzi F, Asslaber M, D’Arrigo C, Vidal-Vanaclocha F, Zavadova E, Chouchane L, Ohashi PS, Hafezi‐Bakhtiari S, Wouters BG, Roehrl M, Nguyen L, Kawakami Y, Hazama S, Okuno K, Ogino S, Gibbs P, Waring P, Sato N, Torigoe T, Itoh K, Patel PS, Shukla SN, Wang Y, Kopetz S, Sinicrope FA, Scripcariu V, Ascierto PA, Marincola FM, Fox BA, Pagès F (2014) Towards the introduction of the ‘Immunoscore’ in the classification of malignant tumours. J Pathol 232:199–209. 10.1002/path.4287 24122236 10.1002/path.4287 PMC4255306

[12] Gasteiger G, Fan X, Dikiy S, Lee SY, Rudensky AY (2015) Tissue residency of innate lymphoid cells in lymphoid and nonlymphoid organs. Science 350:981–985. 10.1126/science.aac9593 26472762 10.1126/science.aac9593 PMC4720139

[13] Głowala-Kosińska M, Chwieduk A, Nieckula J, Saduś-Wojciechowska M, Grosicki S, Rusin A, Nowara E, Giebel S (2013) Association of circulating regulatory T cell number with the incidence and prognosis of diffuse large B-cell lymphoma. Eur J Haematol 91:122–128. 10.1111/ejh.12144 23679234 10.1111/ejh.12144

[14] Jovanovic IP, Pejnovic NN, Radosavljevic GD, Pantic JM, Milovanovic MZ, Arsenijevic NN, Lukic ML (2014) Interleukin-33/ST2 axis promotes breast cancer growth and metastases by facilitating intratumoral accumulation of immunosuppressive and innate lymphoid cells: IL-33 enhances breast cancer progression. Int J Cancer 134:1669–1682. 10.1002/ijc.28481 24105680 10.1002/ijc.28481

[15] Klose CSN, Kiss EA, Schwierzeck V, Ebert K, Hoyler T, d’Hargues Y, Göppert N, Croxford AL, Waisman A, Tanriver Y, Diefenbach A (2013) A T-bet gradient controls the fate and function of CCR6 – RORγt + innate lymphoid cells. Nature 494:261–265. 10.1038/nature11813 23334414 10.1038/nature11813

[16] Li M, Su X, Wang Y, Fan L, Chai J, Li P, Zhao D, Liu Y, Ma J, Wang K, Yan Q, Guo S, Jin B, Liang R, Wang Z (2020) Lineage-negative lymphoma with a helper innate lymphoid cell phenotype. Virchows Arch 476:285–293. 10.1007/s00428-019-02658-x 31522287 10.1007/s00428-019-02658-x

[17] Lordo MR, Scoville SD, Goel A, Yu J, Freud AG, Caligiuri MA, Mundy-Bosse BL (2021) Unraveling the role of innate lymphoid cells in Acute myeloid leukemia. Cancers 13:320. 10.3390/cancers13020320 33477248 10.3390/cancers13020320 PMC7830843

[18] Loyon R, Jary M, Salomé B, Gomez-Cadena A, Galaine J, Kroemer M, Romero P, Trabanelli S, Adotévi O, Borg C, Jandus C (2019) Peripheral innate lymphoid cells are increased in First Line Metastatic Colorectal Carcinoma patients: a negative correlation with Th1 Immune responses. Front Immunol 10:2121. 10.3389/fimmu.2019.02121 31555301 10.3389/fimmu.2019.02121 PMC6742701

[19] Maggi E, Veneziani I, Moretta L, Cosmi L, Annunziato F (2020) Group 2 innate lymphoid cells: a double-edged Sword in Cancer? Cancers 12:3452. 10.3390/cancers12113452 33233582 10.3390/cancers12113452 PMC7699723

[20] Martínez-Lostao L, Anel A, Pardo J (2015) How do cytotoxic lymphocytes kill Cancer cells? Clin. Cancer Res 21:5047–5056. 10.1158/1078-0432.CCR-15-0685 10.1158/1078-0432.CCR-15-0685 26567364

[21] Morvan MG, Lanier LL (2016) NK cells and cancer: you can teach innate cells new tricks. Nat Rev Cancer 16:7–19. 10.1038/nrc.2015.5 26694935 10.1038/nrc.2015.5

[22] Nagasawa M, Heesters BA, Kradolfer CMA, Krabbendam L, Martinez-Gonzalez I, De Bruijn MJW, Golebski K, Hendriks RW, Stadhouders R, Spits H, Bal SM (2019) KLRG1 and NKp46 discriminate subpopulations of human CD117 + CRTH2 – ILCs biased toward ILC2 or ILC3. J Exp Med 216:1762–1776. 10.1084/jem.20190490 31201208 10.1084/jem.20190490 PMC6683990

[23] Ohne Y, Silver JS, Thompson-Snipes L, Collet MA, Blanck JP, Cantarel BL, Copenhaver AM, Humbles AA, Liu Y-J (2016) IL-1 is a critical regulator of group 2 innate lymphoid cell function and plasticity. Nat Immunol 17:646–655. 10.1038/ni.3447 27111142 10.1038/ni.3447

[24] Roma S, Camisaschi C, Mancuso P, Trabanelli S, Vanazzi A, Villa S, Prati D, Fiori S, Lorenzini D, Tabanelli V, Pileri S, Tarella C, Jandus C, Bertolini F (2022) Dampening of cytotoxic innate lymphoid cells: a new tumour immune escape mechanism in B cell non-hodgkin’s lymphoma. Cell Immunol 382:104615. 10.1016/j.cellimm.2022.104615 36228388 10.1016/j.cellimm.2022.104615

[25] Ruf B, Greten TF, Korangy F (2023) Innate lymphoid cells and innate-like T cells in cancer — at the crossroads of innate and adaptive immunity. Nat Rev Cancer 23:351–371. 10.1038/s41568-023-00562-w 37081117 10.1038/s41568-023-00562-w

[26] Salimi M, Wang R, Yao X, Li X, Wang X, Hu Y, Chang X, Fan P, Dong T, Ogg G (2018) Activated innate lymphoid cell populations accumulate in human tumour tissues. BMC Cancer 18:341. 10.1186/s12885-018-4262-4 29587679 10.1186/s12885-018-4262-4 PMC5870240

[27] Savage AK, Liang H-E, Locksley RM (2017) The development of steady-state activation hubs between adult LTi ILC3s and primed macrophages in small intestine. J Immunol 199:1912–1922. 10.4049/jimmunol.1700155 28747343 10.4049/jimmunol.1700155 PMC5568484

[28] Sonnenberg GF, Hepworth MR (2019) Functional interactions between innate lymphoid cells and adaptive immunity. Nat Rev Immunol 19:599–613. 10.1038/s41577-019-0194-8 31350531 10.1038/s41577-019-0194-8 PMC6982279

[29] Spits H, Artis D, Colonna M, Diefenbach A, Di Santo JP, Eberl G, Koyasu S, Locksley RM, McKenzie ANJ, Mebius RE, Powrie F, Vivier E (2013) Innate lymphoid cells — a proposal for uniform nomenclature. Nat Rev Immunol 13:145–149. 10.1038/nri3365 23348417 10.1038/nri3365

[30] Susanibar-Adaniya S, Barta SK (2021) 2021 update on diffuse large B cell lymphoma: a review of current data and potential applications on risk stratification and management. Am J Hematol 96:617–629. 10.1002/ajh.26151 33661537 10.1002/ajh.26151 PMC8172085

[31] Tanaka A, Sakaguchi S (2017) Regulatory T cells in cancer immunotherapy. Cell Res 27:109–118. 10.1038/cr.2016.151 27995907 10.1038/cr.2016.151 PMC5223231

[32] Trabanelli S, Curti A, Lecciso M, Salome B, Riether C, Ochsenbein A, Romero P, Jandus C (2015) CD127 + innate lymphoid cells are dysregulated in treatment naive acute myeloid leukemia patients at diagnosis. Haematologica 100:e257–e260. 10.3324/haematol.2014.119602 25710455 10.3324/haematol.2014.119602 PMC4486236

[33] Trabanelli S, Gomez-Cadena A, Salomé B, Michaud K, Mavilio D, Landis BN, Jandus P, Jandus C (2018) Human innate lymphoid cells (ILCs): toward a uniform immune-phenotyping: hILC PHENOTYPE. Cytometry B Clin Cytom 94:392–399. 10.1002/cyto.b.21614 29244250 10.1002/cyto.b.21614

[34] Verrier T, Satoh-Takayama N, Serafini N, Marie S, Di Santo JP, Vosshenrich CAJ (2016) Phenotypic and functional plasticity of murine intestinal NKp46 + group 3 innate lymphoid cells. J Immunol 196:4731–4738. 10.4049/jimmunol.1502673 27183613 10.4049/jimmunol.1502673

[35] von Burg N, Chappaz S, Baerenwaldt A, Horvath E, Bose Dasgupta S, Ashok D, Pieters J, Tacchini-Cottier F, Rolink A, Acha-Orbea H, Finke D (2014) Activated group 3 innate lymphoid cells promote T-cell–mediated immune responses. Proc Natl Acad Sci 111:12835–12840. 10.1073/pnas.1406908111 25136120 10.1073/pnas.1406908111 PMC4156721

[36] Warner K, Ghaedi M, Chung DC, Jacquelot N, Ohashi PS (2022) Innate lymphoid cells in early tumor development. Front Immunol 13:948358. 10.3389/fimmu.2022.948358 36032129 10.3389/fimmu.2022.948358 PMC9411809

[37] Withers DR, Hepworth MR (2017) Group 3 innate lymphoid cells: communications hubs of the Intestinal Immune System. Front Immunol 8:1298. 10.3389/fimmu.2017.01298 29085366 10.3389/fimmu.2017.01298 PMC5649144

[38] Wu Y, Yan Y, Su Z, Bie Q, Chen X, Barnie PA, Guo Q, Wang S, Xu H (2017) Enhanced circulating ILC2s and MDSCs may contribute to ensure maintenance of Th2 predominant in patients with lung cancer. Mol Med Rep 15:4374–4381. 10.3892/mmr.2017.6537 28487978 10.3892/mmr.2017.6537

[39] Zhao H, Wu L, Yan G, Chen Y, Zhou M, Wu Y, Li Y (2021) Inflammation and tumor progression: signaling pathways and targeted intervention. Signal Transduct Target Ther 6:263. 10.1038/s41392-021-00658-5 34248142 10.1038/s41392-021-00658-5 PMC8273155

